# Nitric oxide as a mechanistic link between metabolic dysfunction and cognitive decline in type 2 diabetes

**DOI:** 10.1042/BST20250325

**Published:** 2026-09-16

**Authors:** Ana L. Marçal, João Laranjinha, Cátia F. Lourenço

**Affiliations:** 1 Center for Neuroscience and Cell Biology, University of Coimbra, Coimbra, Portugal; 2 Faculty of Pharmacy, University of Coimbra, Coimbra, Portugal; 3 Centre for Innovative Biomedicine and Biotechnology, Coimbra, Portugal

**Keywords:** Cerebrovascular dysfunction, Neurovascular coupling, Nitric oxide, Type 2 diabetes mellitus

## Abstract

Type 2 diabetes mellitus (T2DM) is increasingly recognised as a driver of cerebrovascular dysfunction and cognitive decline, yet the molecular mechanisms underlying this association remain incompletely understood. Here, we review and support a key mechanistic role for nitric oxide (^•^NO), a pleiotropic diffusible messenger that integrates vascular, neuronal, and metabolic functions within the neurovascular unit (NVU). Under physiological conditions, ^•^NO integrates vascular and metabolic responses by coupling blood supply to neuronal activity and fine-tuning cellular energy metabolism. In T2DM, chronic hyperglycemia and insulin resistance converge to reduce ^•^NO bioavailability through multiple mechanisms: mitochondrial and NADPH oxidase-derived superoxide production, endothelial nitric oxide synthases (eNOS) uncoupling via BH_4_ oxidation, and impaired PI3K-Akt-dependent eNOS activation. The resulting redox imbalance shifts the cerebrovascular environment towards oxidant-mediated damage, compromising the functional and structural integrity of the NVU. This translates into impaired cerebral blood flow regulation and maladaptive remodelling of the cerebrovascular network, ultimately disrupting neurovascular coupling (NVC) and reducing regional cerebral perfusion. Together, impaired perfusion and disrupted NVC result in a sustained mismatch between energy supply and neuronal demand, particularly in metabolically vulnerable regions such as the hippocampus, ultimately leading to progressive cognitive impairment. In sum, the present review integrates current mechanistic evidence positioning ^•^NO dysregulation as a central driver of neurovascular and metabolic dysfunction in T2DM, linking impaired cerebral perfusion, disrupted NVC, and structural vascular remodelling to cognitive decline.

## Introduction

Diabetes mellitus, nowadays, represents a major and growing public health challenge. Estimates calculate approximately 588 million individuals living with diabetes worldwide, significantly impacting both individual health and healthcare systems [1]. Among the different forms of the disease, type 2 diabetes mellitus (T2DM) accounts for nearly 90% of cases and is characterised by chronic hyperglycemia and insulin resistance (IR) [1]. Traditionally viewed as peripheral disorders, diabetes mellitus and IR are now recognised as systemic problems, affecting the brain as well [2,3]. Despite its small size, the brain is the most energy-consuming organ in the human body, accounting for ∼20% of the energy produced at rest, yet it has minimal energy reserves [4]. This high metabolic demand emphasises the critical role of cerebral blood flow (CBF) in meeting the brain’s energy requirements, essential for synaptic transmission, neuroplasticity, and overall neuronal performance [5].

The regulation of cerebral blood perfusion is choreographed by the neurovascular unit (NVU), a cooperative structural and functional assembly composed of neurons, astrocytes, endothelial cells (ECs), smooth muscle cells (SMCs), and pericytes [6]. Within this system, neurons initiate activity-dependent signals, astrocytes amplify and pass metabolic information, ECs regulate vascular tone and barrier integrity, and pericytes control capillary diameter and microvascular flow [7,8]. Through these coordinated interactions, neuronal metabolic demand is translated into vascular responses that adjust blood supply to neuronal activity, a process known as neurovascular coupling (NVC) [6]. While NVC represents a key component of cerebral microvascular regulation, specifically representing the rapid increase in regional CBF in response to neuronal activation, in a broader sense cerebral microvascular regulation also includes processes that maintain vascular homeostasis within the brain, including control of basal vascular tone and the maintenance of the blood–brain barrier (BBB) [4]. The NVU’s capacity to integrate neuronal, glial, and vascular signals is essential for maintaining metabolic and vascular stability in brain physiology. Among the molecular mediators taking part in these processes, nitric oxide (^•^NO) has been identified as a central player [9–11]. Due to its ability to rapidly diffuse across cellular membranes, ^•^NO is in a unique position to coordinate the tight connection between the NVU components [12,13]. Accumulating evidence associates T2DM and IR with changes in CBF and cognitive function [14–16]. While the pathophysiology of diabetic cognitive decline is multifactorial, encompassing contributions from neuroinflammation, impaired glucose metabolism, and synaptic dysfunction, among others, in the present review we focus on ^•^NO as a central mechanistic link through which T2DM and its hallmarks (hyperglycemia and IR) converge to disrupt cerebrovascular function, positioning ^•^NO not as an exclusive cause but as a key integrative node within this complex disease landscape.

## Nitric oxide signalling within the neurovascular unit

^•^NO is a pleiotropic free radical and highly diffusible paracrine messenger that operates through volume signalling to integrate neuronal, vascular, and metabolic processes within the brain [12]. Its unique physicochemical characteristics enable it to diffuse across biological membranes, making it an important regulator of cerebral homeostasis [17]. In the brain, ^•^NO production involves a family of nitric oxide synthases (NOS) [18] that enzymatically convert Larginine into L-citrulline and ^•^NO, requiring oxygen and cofactors, including NADPH and tetrahydrobiopterin (BH_4_). Among these enzymes, the endothelial nitric oxide synthase (eNOS) and neuronal nitric oxide synthase (nNOS) are constitutively expressed, contrasting with the inducible nitric oxide synthase, which is primarily regulated at the transcriptional level, being expressed in immune and other cell types in response to inflammatory stimuli [19]. nNOS is the most abundant isoform in the central nervous system, mainly expressed in neurons where it is often tethered to the *N*-methyl-D-aspartate (NMDA) glutamate receptor via the scaffolding protein postsynaptic density protein-95 [20]. This arrangement allows nNOS to translate synaptic activity into rapid ^•^NO production. During synaptic activity, the glutamate released into the synaptic cleft binds to postsynaptic NMDA receptors, eliciting a rapid influx of Ca^2+^ activates nNOS via calmodulin, leading to ^•^NO production [21]. Endothelial NOS is primarily expressed in ECs within the caveolae, forming a complex with caveolin-1 that inhibits its activity [22]. Its activity is modulated through several mechanisms, including shear stress, involving the enzyme’s dissociation from its interaction with caveolin-1, Ca^2+^/calmodulin binding or phosphorylation of specific serine residues (e.g. Ser^1177^) via PI3K-Akt pathway [22]. Recent evidence also indicates that group 1 metabotropic glutamate receptors (mGluR1/5) are expressed on human brain microvascular ECs and can act as sensors on neuronal activity, triggering Ca^2+^ dependent eNOS activation [23].

**Figure 1:**
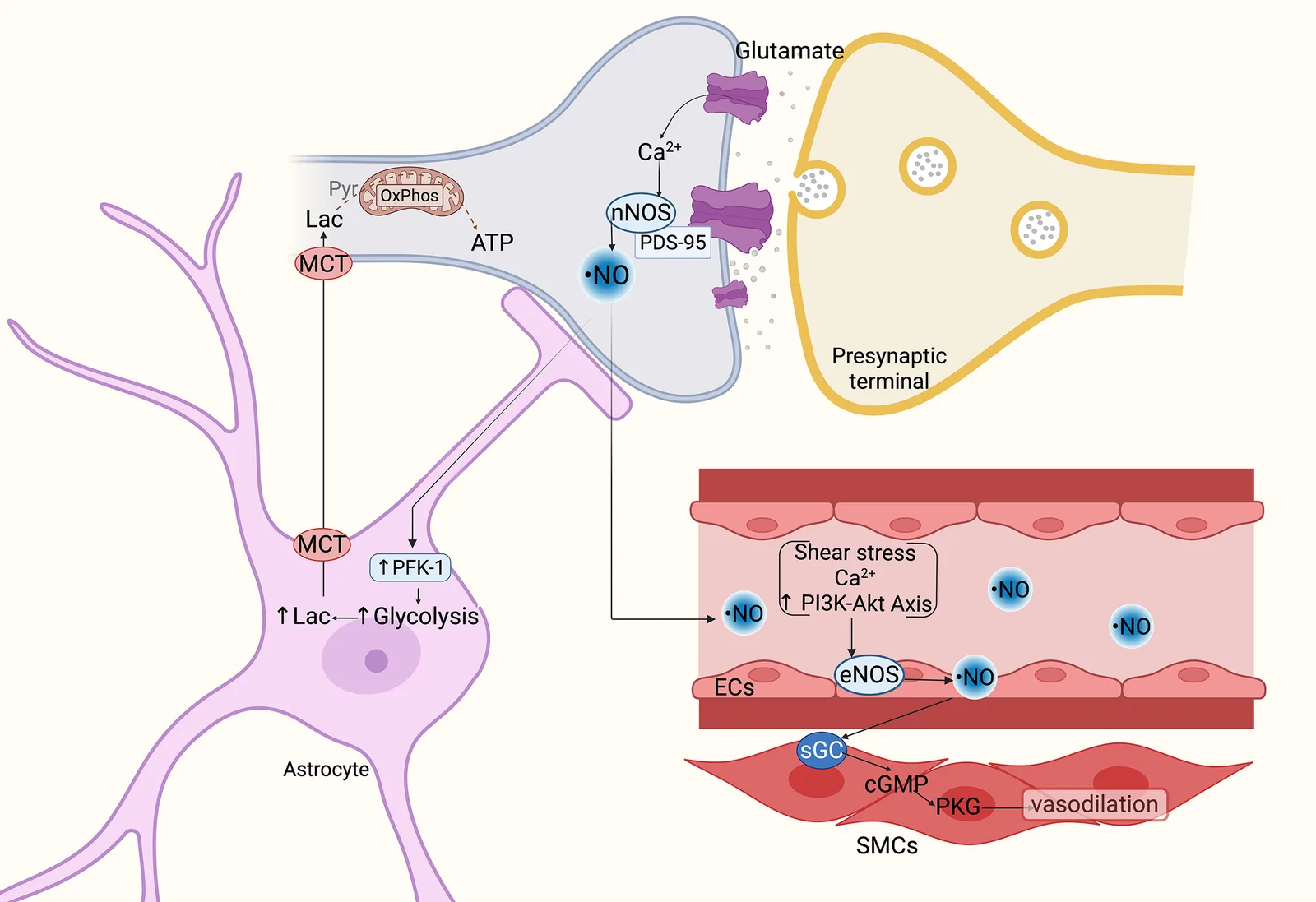
Nitric oxide-mediated neurovascular coupling and astrocyte–neuron metabolic support in the healthy NVC Neuronal activity induces Ca^2+^-dependent nNOS activation and ^•^NO production, which diffuses to nearby vessels to promote vasodilation via the sGC-cGMP-PKG pathway. Endothelial ^•^NO, generated through shear stress and PI3K-Akt signalling, further contributes to vascular tone regulation. In parallel, ^•^NO modulates astrocytic glycolysis and lactate shuttling to neurons, supporting mitochondrial ATP production and matching energy supply to neuronal demand.

A prominent physiological role of ^•^NO in the brain is the regulation of CBF (Figure 1). ^•^NO synthesised by neurons and ECs readily diffuses to reach nearby vascular SMCs in arteriolar walls, where it activates soluble guanylate cyclase (sGC) and leads to the formation of cyclic guanosine monophosphate (cGMP). Elevated levels of cGMP activate protein kinase G, leading to smooth muscle relaxation and vasodilation, thereby regulating changes in vascular tone and cerebral blood perfusion [24]. In addition to regulating basal vascular tone, ^•^NO also critically contributes to activity-dependent adjustments in CBF during neuronal activity [9]. Its production, tightly coupled to neuronal activity, together with its rapid diffusion, enables efficient matching of CBF to the metabolic demands imposed by neuronal activation. Accordingly, nNOS is recognised as a primary driver of the NVC, with its selective inhibition reducing the hemodynamic response by 64%, exceeding the effect of any other individual target [10]. While it has been classically implicated in the initial rapid phase, recent evidence suggests that this contribution is context-dependent and may not be universal across experimental conditions [25]. In turn, endothelial-derived ^•^NO contributes to the regulation of vascular tone and to NVC by facilitating retrograde dilation from microvessels to upstream pial arteries [26]. In contrast, feedback signalling mechanisms contribute to the maintenance of vascular supply during prolonged neuronal activity [27,28].

Beyond its role in vascular regulation, ^•^NO also acts as a metabolic regulator, helping to coordinate energy supply with neuronal activity. In astrocytes, ^•^NO enhances glycolysis by increasing phosphofructokinase-1 activity via fructose-2,6-bisphosphate, promoting lactate production that is subsequently shuttled to neurons to sustain oxidative phosphorylation [29,30]. Simultaneously, ^•^NO reversibly competes with O_2_ at cytochrome c oxidase, modulating mitochondrial respiration and influencing the balance between oxidative phosphorylation and glycolysis [31]. Through these actions, ^•^NO coordinates neuronal energy demand with vascular supply and substrate utilisation. In addition, ^•^NO can regulate protein function via S-nitrosation of proteins, a reversible reaction in which ^•^NO modifies cysteine thiols to form S-nitrosothiols, thereby modulating the activity of different proteins involved in cellular signalling [17].

Taken together, these mechanisms illustrate the multifaceted role of ^•^NO in the brain, acting simultaneously as a regulator of vascular tone, cellular metabolism and intracellular signalling.

## Nitric oxide dysregulation and functional cerebrovascular impairment

**Figure 2:**
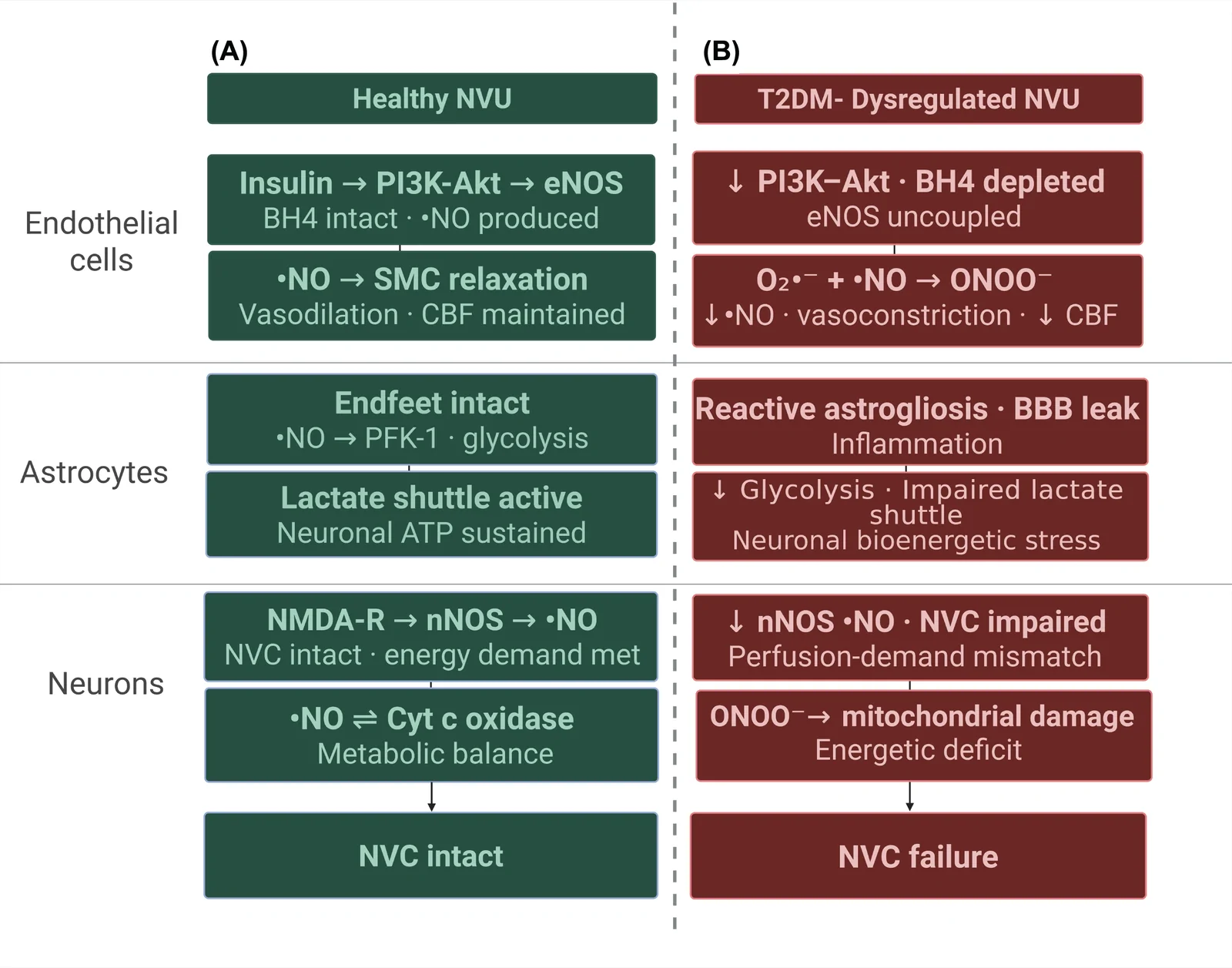
Shift of ^•^NO-dependent neurovascular function in T2DM In the healthy neurovascular unit (**A**), ^•^NO supports endothelial function, astrocytic metabolism, and neuronal signalling, maintaining NVC and adequate cerebral blood flow. In T2DM (**B**), reduced ^•^NO bioavailability and increased nitro-oxidative stress disrupt these processes, leading to endothelial dysfunction, impaired astrocyte-neuron metabolic coupling, mitochondrial dysfunction, and ultimately neurovascular uncoupling and bioenergetic deficit.

In the diabetic brain, metabolic stress arising from chronic hyperglycemia and redox imbalance disrupts ^•^NO homeostasis, shifting it from physiological regulation to a state of chronic distress (Figure 2). This transformation may be driven by a series of mechanistic pathways that reduce ^•^NO bioavailability and generate reactive oxygen species (ROS), ultimately compromising the integrity of the NVU and leading to neuronal deficits. Elevated intracellular glucose in neural and vascular cells enhances mitochondrial superoxide (O_2_^•−^) production by increasing electron flux through the electron transport chain, leading to mitochondrial hyperpolarisation and electron leak to molecular oxygen [32]. In addition to mitochondria, the NADPH-oxidase (NOX) family of enzymes is a major source of O_2_^•−^, particularly at the vascular level. Persistent hyperglycemia, through increased diacylglycerol formation, and advanced glycation endproducts (AGEs), through RAGE signalling, trigger protein kinase C activation that, in turn, promotes NOX activation by phosphorylating its regulatory subunits, leading to O_2_^•−^ production [32]. The overproduction of O_2_^•−^ leads to the rapid inactivation of ^•^NO because these two free radicals rapidly react at near diffusion-controlled rates to form peroxynitrite (ONOO⁻), a highly reactive molecule that redirects ^•^NO bioactivity towards oxidative and nitrative chemistries [33]. Peroxynitrite promotes irreversible tyrosine nitration of proteins, forming 3-nitrotyrosine, a widely used marker of nitroxidative stress that is elevated in the brains of diabetic animals [34]. In parallel, ONOO⁻ and its reactive intermediaries (^•^OH and ^•^NO_2_) promote oxidative damage to DNA, lipids, and proteins that compromises cellular integrity. For instance, ONOO⁻-mediated nitration of cytoskeletal proteins such as actin is particularly detrimental in the cerebrovasculature, as they contribute to loss of myogenic tone [35]. ^•^NO bioavailability is further compromised by reduced ^•^NO synthesis due to NOS uncoupling. Excessive ROS impairs eNOS activity by oxidising BH_4_, an essential cofactor for eNOS coupling. The reduced availability of BH_4_ shifts eNOS function from ^•^NO production to the generation of O_2_^•−^, thereby establishing a feed-forward cycle of oxidative distress that further limits ^•^NO bioavailability and exacerbates nitroxidative damage [36]. This redox-dependent shift in NOS function also helps to explain why global pharmacological manipulation of the NOS/NO pathway may generate outcomes difficult to interpret. For instance, in STZ-induced diabetic rats, both L-NAME and L-arginine impaired active avoidance performance, despite complex effects on nitrite levels, lipid peroxidation, body weight, and glucose [37]. These findings reinforce the concept that therapeutic strategies should not aim simply to increase or inhibit global NOS activity, but rather to restore NOS coupling, redox balance, and compartmentalised ^•^NO bioavailability.

Not only hyperglycemia, but also IR contributes to decreased ^•^NO bioavailability. In an insulin-sensitive state, insulin binds its receptor, activating the PI3K-Akt pathway leading to eNOS phosphorylation and activation. This pathway plays an important role in maintaining endothelial function and vascular homeostasis. However, IR in T2DM down-regulates the PI3K pathway, thereby reducing eNOS phosphorylation and ^•^NO production [38]. In parallel, insulin signalling through the mitogen-activated protein kinase (MAPK) pathway remains preserved, leading to the production of endothelin-1, a potent vasoconstrictor. This imbalance between reduced PI3K-Akt signalling and enhanced MAPK activity tilts the vascular environment from ^•^NO-mediated vasodilation towards increased vasoconstrictive signalling, contributing to compromised cerebral perfusion in IR [36]. A further endogenous mechanism contributing to reduced ^•^NO bioavailability in T2DM is the accumulation of asymmetric dimethylarginine (ADMA), a methylated derivative of L-arginine that competitively inhibits all NOS isoforms. Under physiological conditions, ADMA is primarily catabolised by dimethylarginine dimethylaminohydrolase (DDAH), which exists as two isoforms: DDAH1 is mainly associated with tissues expressing nNOS, including the brain, whereas DDAH2 is enriched in tissues expressing eNOS, including ECs. Importantly, DDAH activity is highly sensitive to oxidative inactivation, as excessive ROS can oxidise the catalytic cysteine residue within its active site, thereby impairing ADMA degradation [39]. Consistently, circulating ADMA levels have been reported to be increased in patients with T2DM [40,41]

Beyond its vascular effects, reduced ^•^NO bioavailability in T2DM also compromises its metabolic support functions within the NVU. Under physiological conditions, eNOS-produced ^•^NO stabilises HIF-1α in astrocytes, boosting glycolytic enzymes and the lactate transporter MCT-4, which promotes lactate production for neuronal use [42]. This regulation occurs at nanomolar levels with rapid, reversible signalling [43]. Therefore, even modest decreases in eNOS activity can impair astrocytic glycolysis and disrupt the lactate shuttle between astrocytes and neurons. At the same time, the nitroxidative environment shifts mitochondrial regulation from reversible ^•^NO effects on cytochrome c oxidase to irreversible damage caused by peroxynitrite. While astrocytes adapt by increasing glycolysis, neurons, which cannot do so, face bioenergetic crises, increasing neuronal vulnerability beyond vascular issues alone [44].

## Structural remodelling of vasculature in the diabetic brain

The vascular dysfunction in T2DM extends beyond functional impairment, being further associated with structural alterations. One of the earliest and most prominent features of this structural deterioration is microvascular rarefaction, characterised by reduced vascular density and loss of functional capillaries [15,45]. Advanced 3D imaging demonstrated a significant reduction in the percentage area of perfused vessels across different brain regions [45]. Parallel comparisons in rodent models reveal that T2DM triggers a measurable decrease in vascular density in critical regions, such as the striatum and hippocampus, and, coupled with this reduced density, they showed that there was also reduced CBF [15]. In diabetic animals, hippocampal vascular density has been reported to decrease by approximately 8.73%, reflecting progressive microvascular loss [15]. These microstructural alterations might create localised perfusion dysregulation that contributes to impaired NVC [46,47].

In addition to microvascular rarefaction, diabetes is associated with alterations in vascular architecture, including increased tortuosity and disrupted branching patterns. In the retina, often viewed as a ‘window to the brain’ due to shared anatomical and physiological features [48], numerous studies consistently demonstrate increased vessel tortuosity and altered branching geometry as early features of diabetic microangiopathy [49–51]. Although evidence in the brain is comparatively limited, similar alterations have been described in the cerebral microvasculature. Studies in diabetic models reveal that microvascular hypoperfusion is detectable as early as four weeks of age. In adult diabetic mice, newly formed vessels exhibit dysfunctional loop structures rather than typical terminal points, a maladaptive pattern that contributes to poor tissue formation [52]. This architectural decline is linked to a profound remodelling of the gliovascular unit, characterised by pericyte loss and hyperactive astrogliosis with finer processes wrapping around cerebral vessels [53]. Molecular analysis reveals that these structural shifts are driven by reduced PPARy expression and activation of the NLRP3 inflammasome, which elevates inflammatory cytokines such as IL-1β and TNF-α [52,53]. Furthermore, BBB dysfunction is evidenced by either overt IgG leakage into the brain parenchyma or the specific accumulation of IgG on the luminal side of microvascular walls [53,54]. Importantly, early pharmacological interventions with glycemic control agents such as metformin or pioglitazone can mitigate this dysfunctional remodelling and suppress the underlying chronic neuroinflammation [53,54].

While vascular remodelling is a recognised feature of T2D, the molecular mechanisms driving these alterations are not fully elucidated. However, a prominent role has been attributed to redox-dependent alterations of the extracellular matrix (ECM). Alterations in ^•^NO bioavailability and excessive production of RONS can disrupt the balance between ECM synthesis and degradation, promoting a shift towards pathological ECM accumulation. Hyperglycemia and redox imbalance activate transforming growth factor-β (TGF-β) signalling which leads to SMAD2/3-dependent transcription of ECM components, including collagen IV, laminin, and fibronectin, contributing to basement membrane thickening [55]. In parallel, oxidative modifications and glycation of matrix proteins enhance cross-linking, further increasing matrix stiffness and resistance to degradation [56]. Concomitantly, dysregulation of matrix metalloproteinases (MMPs), particularly MMP-2 and MMP-9, contributes to structural instability of microvasculature by degrading key components of the vascular basement membrane. These enzymes, present in ECs, astrocytes, and microglia, are tightly regulated by redox signalling mechanisms, both at the transcription and post-translational levels. Reflecting its dual role under different redox conditions, ^•^NO thought to regulate MMPs in a biphasic manner: At physiological settings, ^•^NO inhibits MMP-9 expression and activity via cGMPdependent pathways, while under oxidative stress conditions, it contributes to increased MMPs activation via ONOO^−^-induced oxidative modification of the cysteine residue within the ‘cysteine switch’ of pro-MMPs [57,58]. In addition to enhancing MMP activity, nitroxidative stress also promote the up-regulation of MMPs’ expression via redox-sensitive pathways, including NF-κB and AP-1 activation, and impairs endogenous regulatory mechanisms via the tissue inhibitors of metalloproteinases oxidative-inactivation [58]. The combined effects of increased MMP expression, direct enzymatic activation, and impaired inhibition drive uncontrolled ECM remodelling, leading to microvascular instability. In parallel with TGF-β-mediated matrix deposition, this results in maladaptive remodelling characterised by concurrent matrix accumulation and degradation, ultimately disrupting vascular architecture and neurovascular function.

A further process to be considered is that angiogenesis and neovascularisation are also compromised. While T2DM can increase the risk of cerebral ischemia and hypoperfusion and trigger the up-regulation of vascular endothelial factor to initiate capillary formation, the resulting neovascularisation is mainly pathological and aberrant [59]. The newly formed vessels are poorly formed, structurally fragile, and prone to premature regression, failing to restore perfusion to hypoxic areas. These aberrant vascular patterns may reflect impaired ^•^NO-dependent regulation of angiogenesis and vessel maturation, as ^•^NO is known to modulate endothelial migration, branching, and stabilisation of newly formed vessels [60].

The loss and dysfunction of pericytes is another key driver of the structural failure in diabetic cerebrovasculature. These cells are essential in the NVU, positioned between ECs, astrocytes, and neurons; they coordinate and process signals from their neighbouring cells to generate diverse functional responses, including regulation of the BBB permeability, angiogenesis, and capillary hemodynamic responses [61]. Pericytes are particularly vulnerable to hyperglycemia-induced redox stress, which drives apoptosis via ROS production and AGE-RAGE signalling [62]. This leads to reduced pericyte coverage of cerebral microvessels, while surviving cells exhibit functional impairment [63]. As contractile regulators of capillary diameter, pericytes are essential for maintaining microvascular tone; however, they may induce contractile states under metabolic and nitrosative stress. Persistent capillary constriction limits effective reperfusion and impairs microvascular flow regulation [64]. In addition, emerging evidence indicates that ^•^NO may modulate pericyte-endothelial communication through extracellular vesicle-mediated signalling, thereby influencing vascular remodelling and revascularisation processes [65]. Although demonstrated in ischemic contexts, these findings support a broader role for ^•^NO in coordinating cellular interactions within the NVU.

In sum, structural remodelling of the diabetic brain vasculature can be grouped into key processes: microvascular rarefaction, marked by reduced capillary density and perfusion; architectural and gliovascular alterations, including vessel tortuosity, abnormal branching, inflammation, and BBB disruption; ECM and redox-driven remodelling, where ^•^NO imbalance and oxidative distress promote both matrix accumulation and degradation; impaired angiogenesis, leading to fragile, dysfunctional vessels; and pericyte loss and dysfunction, which compromises vascular stability and flow regulation. Together, these changes drive neurovascular impairment in type 2 diabetes.

## From cerebrovascular dysfunction to cognitive decline

The structural and molecular alterations discussed above, and compiled in Figure 2, converge functionally at the level of impaired NVC and cerebral perfusion, which ultimately translates into cognitive decline. In patients with T2DM, the hemodynamic response function becomes significantly slower, characterised by a higher peak latency and lower peak amplitude across all brain regions. Detailed analysis reveals a pronounced initial dip, suggesting that the brain’s initial avidity for oxygen is not compensated for by a timely blood supply, and a less prominent but longer-lasting undershoot [66]. In clinical settings, this failure is often detected by transcranial Doppler as a reduction in the ‘natural frequency’, a parameter that reflects the oscillatory properties and tone of the cerebrovascular system. Reduced natural frequency serves as a sensitive early marker for NVC dysfunction, occurring even in diabetic and hypertensive subjects without symptomatic cerebrovascular disease [67].

Epidemiological studies consistently demonstrated that patients with diabetes have a significantly higher risk of developing mild cognitive impairment and dementia [68]. For instance, individuals with T2DM are 60% more prone to develop Alzheimer’s disease in comparison with non-diabetic subjects [69]. As previously stated, although the mechanisms linking diabetes and neurodegeneration are multifactorial, increasing evidence suggests that cerebrovascular dysfunction emerges as a central factor and implicates compromised ^•^NO signalling as a key driver. When cerebral perfusion becomes impaired, neuronal activations are no longer matched by adequate increases in local perfusion, resulting in a quantitative and temporal neurovascular uncoupling that can lead to chronic energetic stress within neuronal circuits. In T2DM, this stress is compounded by a 31% reduction in the maximum glucose transport rate and impaired glycogen metabolism, which hampers the availability of energy for synaptic plasticity [70]. Certain brain regions appear particularly vulnerable to diabetes-associated cerebrovascular dysfunction, reflecting their high metabolic demand and tight dependence on finely tuned NVC [71,72]. In a rodent model of T2DM, impaired hippocampal NVC has been demonstrated as an early event, occurring in close association with disrupted glutamate-evoked ^•^NO dynamics and deficits in spatial learning and memory performance [73]. In the same animal model, it was also reported marked cerebrovascular remodelling, characterised by increased cerebral vascular density, together with a higher proportion of immature/non-perfused microvessels [74].Consistent with these findings, reduced regional CBF in the hippocampus has been associated with poorer memory performance in humans [75]. Additionally, impaired CBF in the cerebellar network has been linked to both gait alterations and executive processing deficits [76]. Importantly, this vulnerability is not restricted to individual regions but involves large-scale brain networks. Hypoperfusion within the default mode network, particularly in the precuneus and posterior cingulate cortex, is strongly associated with deficits in executive function, attention, and information processing speed [76]. Persistent neurovascular dysfunction ultimately contributes to structural brain damage, manifested as cerebral small vessel disease (CSVD). Imaging markers, such as white matter hyperintensities, lacunes, and enlarged perivascular spaces in the basal ganglia, are significantly more prevalent in patients with T2DM [77]. Research indicates that a moderate or severe total CSVD burden is a risk factor for mild cognitive impairment. These structural abnormalities reflect end-stage parenchymal damage stemming from endothelial dysfunction and increased BBB. This increased permeability allows plasma constituents to leak into the perivascular space, which may directly damage neurons, trigger neuroinflammation, and disrupt the neural connectivity necessary for cognitive stability [77,78]. Ultimately, the progression from cerebrovascular dysfunction to cognitive decline follows the ‘ticking clock hypothesis’, suggesting that microvascular disease processes often begin during the prediabetic stage, long before the clinical onset of diabetes [78]. This cumulative evidence supports a model in which early functional failures gradually progress to manifest structural lesions, ultimately resulting in clear deficits in memory and executive control.

## Conclusion and future perspectives

T2DM is firmly recognised as a driver of cerebrovascular dysfunction and a risk factor for the development of cognitive decline. Within this framework, ^•^NO emerges as a key integrator of neuronal signalling, vascular tone, and metabolic processes, required to sustain cerebral blood perfusion and brain homeostasis.

Disruption of ^•^NO signalling constitutes a critical mechanistic link between metabolic and cerebrovascular dysfunction. Persistent hyperglycemia, oxidative stress, and IR converge to limit ^•^NO bioavailability, leading to structural and functional impairment of the cerebral vasculature compromising cerebral perfusion and increasing the vulnerability of metabolically demanding brain regions, such as the hippocampus, thereby contributing to the cognitive deficits frequently observed in T2DM patients.

Although progress has been made, multiple crucial questions remain unanswered. One important challenge is to clarify the temporal dynamics of ^•^NO-mediated dysfunction during the disease progression. Emerging longitudinal evidence suggests that neurovascular impairment develops early in T2DM patients [16] and animal models [73], yet the point at which these alterations become irreversible remains poorly defined. In parallel, increasing evidence indicates that neurovascular impairment may manifest heterogeneously across brain regions [79], suggesting the existence of tissue-specific mechanisms that determine regional vulnerability. Understanding how differences in endothelial signalling, oxidative distress responses, and local metabolic regulation shape the susceptibility of specific vascular networks will be essential for explaining selective neurovascular dysfunction and its contribution to diabetes-associated cognitive decline. Beyond mechanistic understanding, the framework outlined in the present review has direct therapeutic implications. Strategies aimed at restoring ^•^NO bioavailability represent a rational approach to preserving neurovascular function and preventing cognitive decline in T2DM. Among non-pharmacological interventions, inorganic dietary nitrate supplementation has attracted considerable attention, as it can replenish ^•^NO pools through the nitrate-nitrite-NO pathway, even under conditions of NOS dysfunction [80,81]. Phosphodiesterase type 5 (PDE5) inhibitors, such as sildenafil, represent a complementary pharmacological approach that amplifies ^•^NO-mediated signalling downstream of its synthesis by preventing cGMP degradation. Experimental evidence suggests that PDE5 inhibition can attenuate neuroinflammation [82] and cognitive dysfunction [83] and improve cerebrovascular function [84]. Additional promising strategies include restoring eNOS coupling through BH_4_ supplementation and antioxidant approaches aimed at limiting superoxide-mediated ^•^NO inactivation, particularly NOX inhibition and mitochondria-targeted antioxidants [85–87]. Addressing these unresolved issues may uncover new strategies to preserve ^•^NO signalling and sustain neurovascular homeostasis in metabolic disease.

## Perspectives

- Cerebrovascular dysfunction is a key driver of cognitive decline in T2DM, shifting the focus towards neurovascular mechanisms. ^•^NO sits at the interface of vascular, neuronal, and metabolic regulation within the neurovascular unit.
- T2DM reduces ^•^NO bioavailability and promotes nitro-oxidative stress, impairing NVC, cerebral blood flow, and metabolic support. This leads to a mismatch between energy supply and demand, compounded by vascular remodelling and culminating in cognitive decline.
- A major challenge is to determine whether restoring ^•^NO bioavailability can effectively reverse early neurovascular dysfunction in T2DM. This will require targeted and stage-specific therapeutic approaches.

## Open Access

This article has been published open access under our Subscribe to Open programme, made possible through the support of our subscribing institutions, learn more here: https://portlandpress.com/pages/open_access_options_and_prices#conditional.

## Abbreviations

AGEs: advanced glycation endproducts
ADMA: asymmetric dimethylarginine
BBB: blood–brain barrier
BH_4_: tetrahydrobiopterin
CBF: cerebral blood flow
cGMP: cyclic guanosine monophosphate
CSVD: cerebral small vessel disease
DDAH: dimethylarginine dimethylaminohydrolase
ECs: endothelial cells
ECM: extracellular matrix
eNOS: endothelial nitric oxide synthase
IgG: immunoglobulin G
IR: insulin resistance
L-NAME: Nω-nitro-L-arginine methyl ester
MAPK: mitogen-activated protein kinase
MMPs: matrix metalloproteinases
NF-κB: nuclear factor kappa B
NMDA: *N*-methyl-D-aspartate
nNOS: neuronal nitric oxide synthase
•NO: nitric oxide
NOX: NADPH oxidase
NOS: nitric oxide synthases
NVC: neurovascular coupling
NVU: neurovascular unit
PDE5: phosphodiesterase type 5
RAGE: receptor for advanced glycation end products
ROS: reactive oxygen species
sGC: soluble guanylate cyclase
SMCs: smooth muscle cells
TGF-β: transforming growth factor-β
T2DM: type 2 diabetes mellitus
